# Utilizing Friction Energy on Nanoflowers (Zinc Oxide and Zinc Oxide/Neodymium Oxide) for Tribocatalysis of Doxycycline

**DOI:** 10.3390/molecules30234653

**Published:** 2025-12-04

**Authors:** Dobrina Ivanova, Hristo Kolev, Ralitsa Mladenova, Yordanka Karakirova, Nina Kaneva

**Affiliations:** 1 Laboratory of Nanoparticle Science and Technology, Department of General and Inorganic Chemistry, Faculty of Chemistry and Pharmacy, University of Sofia, 1 James Bourchier Blvd., 1164 Sofia, Bulgaria; dobrina.k.ivanova@gmail.com; 2 Institute of Catalysis, Bulgarian Academy of Sciences, “Acad. G. Bonchev” Str., Bldg. 11, 1113 Sofia, Bulgaria; hgkolev@ic.bas.bg (H.K.); ralitsa@ic.bas.bg (R.M.); daniepr@ic.bas.bg (Y.K.)

**Keywords:** tribocatalysis, doxycycline, rare-earth oxides, ZnO nanoflowers

## Abstract

Mechanical energy is a plentiful, environmentally friendly, and sustainable energy source in the natural world. In this work, we successfully use friction to transform mechanical energy into ZnO and ZnO/Nd_2_O_3_ (1, 2, 3, 4 and 5 mol%) tribocatalysts. Under magnetic stirring, the catalyst particles and the polytetrafluoroethylene (PTFE)-sealed magnetic bar rubbed against one another, transferring electrons across the contact interface. While the PTFE absorbed the electrons, holes were simultaneously left on the catalyst. Because of their potent oxidative power, the holes in the valence band of sol–gel catalysts can efficiently oxidize organic pollutants, much like photocatalysis. In the absence of light, the tribocatalytic tests showed that ZnO and ZnO/Nd_2_O_3_ flowers could remove antibiotics (Doxycycline) when magnetized. We could further improve the tribocatalytic performance by adjusting the quantity of rare earth elements (1, 2, 3, 4 and 5 mol%), stirring speed, and magnetic rod type. Besides creating a green tribocatalysis method for organic pollutants’ oxidative purification, this work provides a possible pathway for transforming environmental mechanical energy into chemical energy, which may be applied to environmental remediation and sustainable energy.

## 1. Introduction

The development of numerous advanced oxidation processes (AOPs) for degrading organic pollutants has been driven by the increasing need for sustainable and clean water resources, as well as ongoing pharmaceutical residue contamination in aquatic systems [1,2]. Methods utilizing physical activation sources, such as light, ultrasound, electric, and magnetic fields, have gained significant attention due to their effectiveness in breaking down resistant substances [3,4,5,6]. Since antibiotics are persistent and can increase antibiotic resistance, pharmaceutical contamination is a particular concern. Despite their effectiveness, traditional AOPs like photocatalysis and electrochemical oxidation are rarely feasible in ambient or off-grid environments because they often depend on external energy sources like light or electricity [7,8,9,10,11]. Tribocatalysis and other mechanical energy-driven methods have recently emerged as viable, light-independent alternatives.

Tribocatalysis is a mechanochemical process that produces reactive oxygen species capable of oxidizing organic pollutants through friction-induced charge generation [12,13,14,15,16,17,18]. Its mechanism is similar to mechanochemistry and piezocatalysis, which use mechanical forces to drive redox reactions [19,20]. Zinc oxide, a semiconductor with a wide bandgap and inherent piezoelectric properties, is extensively studied for these applications due to its affordability, chemical stability, and eco-friendliness [21]. Therefore, we believe that ZnO’s triboelectric properties may facilitate pollutant oxidation by converting mechanical energy into chemical energy via friction [22,23,24,25]. Utilizing the frictional properties of nanomaterials for pollutant degradation has thus emerged as an innovative concept. ZnO, a remarkable semiconductor with excellent chemical stability and eco-friendliness, can significantly influence catalytic performance owing to its various morphologies [15]. In reality, nanomaterials with a larger specific surface area display more active sites, which are essential for accelerating organic compound degradation. The tribocatalytic efficiency of zinc oxide can be greatly enhanced through doping with rare-earth elements. These elements improve charge carrier separation, modify surface states, and change the band structure. Neodymium (Nd), which can exist in different oxidation states (Nd^3+^ and Nd^2+^), is a promising dopant that affects the surface chemistry and electronic properties of ZnO-based materials [26,27,28]. Although Nd-doped ZnO shows potential in photocatalytic and luminescent applications, its performance under tribocatalytic conditions remains largely unexplored. Additionally, there is a lack of systematic research on how tribocatalysis influences the surface chemistry of doped ZnO systems, including changes in oxidation states, surface composition, and defect formation.

This study investigates the tribocatalytic degradation of Doxycycline using both pure and ZnO modified with Nd_2_O_3_ nanoflowers (0, 1, 2, 3, 4 and 5 mol%) synthesized via a sol–gel method. The materials are tested under dark stirring conditions to mimic environmental mechanical activation. The effect of Nd_2_O_3_ modification on catalytic activity is related to unchanged surface chemistry and structural features. A comprehensive set of characterization techniques—X-ray photoelectron spectroscopy (XPS), electron paramagnetic resonance (EPR), EPR spin trapping, scanning electron microscopy (SEM), energy-dispersive X-ray spectroscopy (EDS), and UV-vis spectroscopy—is employed to elucidate the relationship between material structure and tribocatalytic performance. This research offers new insights into how rare-earth elements enhance tribocatalysis, demonstrating the potential of Nd_2_O_3_ modified ZnO as an effective catalyst for removing pharmaceutical contaminants from water.

## 2. Results and Discussion

### 2.1. Structural and Morphological Characterization

**Figure 1:**
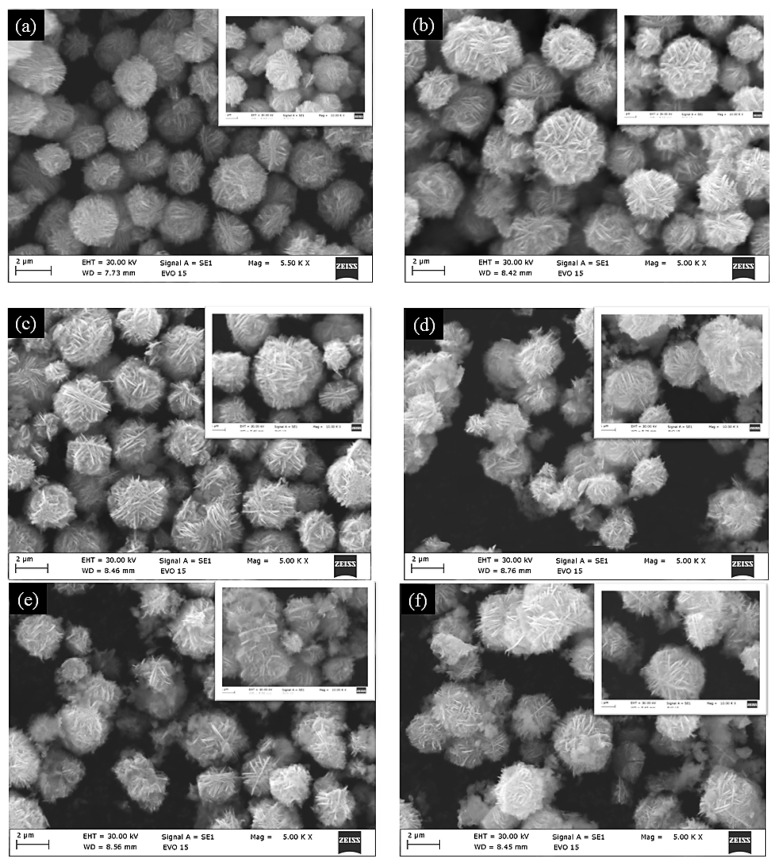
SEM micrographs of (**a**) ZnO, (**b**) ZnO/Nd_2_O_3_ (1 mol%), (**c**) ZnO/Nd_2_O_3_ (2 mol%), (**d**) ZnO/Nd_2_O_3_ (3 mol%), (**e**) ZnO/Nd_2_O_3_ (4 mol%), and (**f**) ZnO/Nd_2_O_3_ (5 mol%) tribocatalysts. The insert show morphology surface of the nanoflowers after tribocatalysis.

The morphology of ZnO modified with Nd_2_O_3_ (0, 1, 2, 3, 4 and 5 mol%) catalysts was investigated in detail using scanning electron microscopy (SEM). As shown in Figure 1, all samples exhibit a well-defined flower-like architecture composed of radially arranged nanosheets, forming uniform microscale spherical aggregates. These nanoflower structures are typically several micrometers in diameter, with individual nanosheets providing a high surface-to-volume ratio, which is advantageous for catalytic processes.

For pristine ZnO, the nanoflowers appear dense and homogeneous. Upon the modification with Nd_2_O_3_, the overall flower-like morphology is retained, and no significant structural collapse or irregular growth is observed. However, a closer inspection indicates that the nanosheets become slightly more open and loosely packed in the ZnO/Nd_2_O_3_ catalysts, particularly at higher Nd_2_O_3_ content. This subtle change in texture may facilitate increased accessibility of active sites and promote improved interaction between the catalyst and solution species during tribocatalysis. The surface structure of the nanoflowers remained generally unchanged after tribocatalytic degradation of Doxycycline (see the inserts of Figure 1).

The preservation of the characteristic ZnO nanoflower morphology, even after modified with Nd_2_O_3_, suggests that the synthesis route is effective in maintaining structural integrity while introducing functional heterojunctions. The hierarchical structure with abundant exposed edges and porous intersheet spaces is expected to contribute to enhanced charge generation and reactive oxygen species (ROS) formation under mechanical excitation.

**Figure 2:**
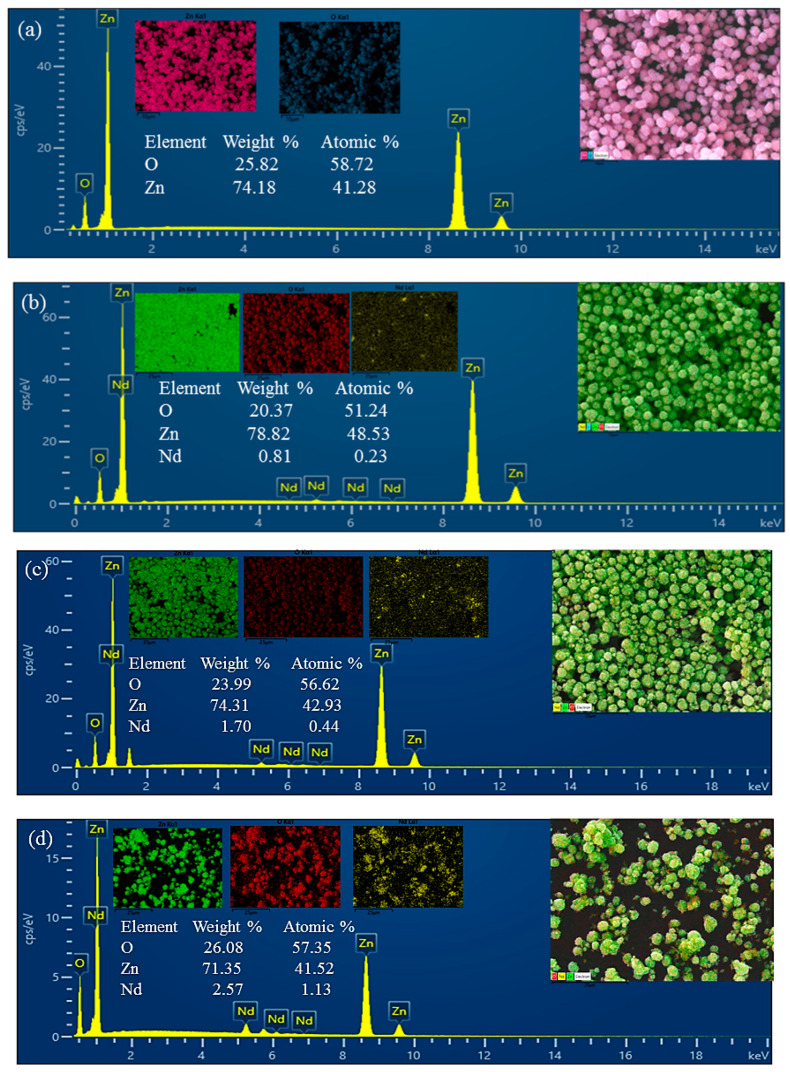

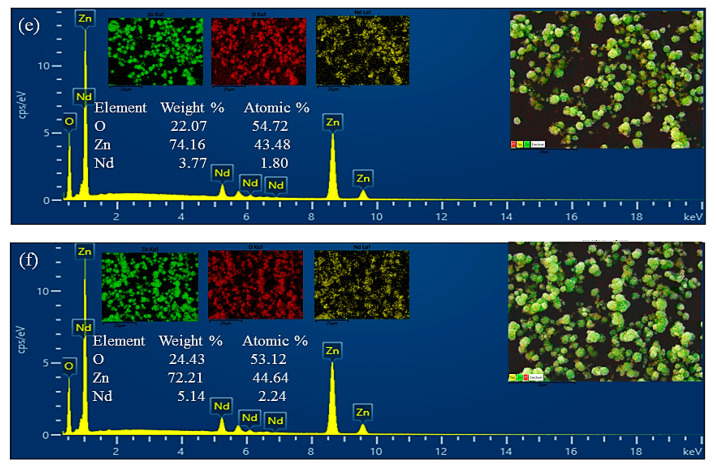
Energy-dispersive X-ray spectroscopy (EDS) spectra of (**a**) pure ZnO, (**b**) ZnO/Nd_2_O_3_ (1 mol%), (**c**) ZnO/Nd_2_O_3_ (2 mol%), and (**d**) ZnO/ Nd_2_O_3_ (3 mol%), (**e**) ZnO/ Nd_2_O_3_ (4 mol%), and (**f**) ZnO/ Nd_2_O_3_ (5 mol%) catalysts.

To verify the chemical composition of the materials, energy-dispersive X-ray spectroscopy (EDS) analysis was performed (Figure 2). The EDS spectra of pristine ZnO confirmed the presence of only Zn and O, without detectable impurities. In the ZnO/Nd_2_O_3_ nanoflowers, additional Nd peaks were clearly observed, confirming the successful introduction of Nd_2_O_3_.

Furthermore, elemental mapping (Figure 2) demonstrated that Zn, O, and Nd are homogeneously distributed throughout the nanoflowers, indicating uniform formation of the composite without significant phase segregation. The combination of SEM morphology and EDS mapping confirms that the ZnO/Nd_2_O_3_ flowers retain their characteristic nanoflower structure while achieving a uniform distribution of Nd_2_O_3_. The hierarchical, porous morphology together with the well-dispersed Nd_2_O_3_ domains is expected to provide abundant active sites and favorable heterojunction interfaces, thereby contributing to the enhanced tribocatalytic activity observed in the degradation of doxycycline.

**Figure 3:**
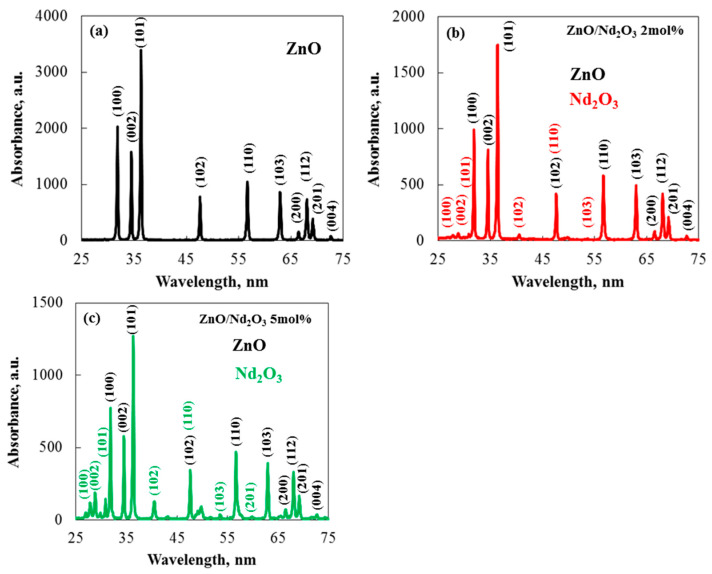
XRD patterns of ZnO modified with Nd_2_O_3_ catalysts prepared by the sol–gel method at neodymium concentrations of 0 (**a**), 2 (**b**) and 5 (**c**) mol%.

The identity, crystallinity, and purity of the as-synthesised samples were verified by XRD analysis. Figure 3 shows the as-synthesised samples’ XRD patterns. The XRD pattern of the synthesized ZnO flowers in Figure 3a shows distinct peaks at 2θ values: 31.77°, 34.48°, 36.22°, 47.59°, 56.63°, 62.80°, 66.45°, 67.89°, 69.12°, 72.46°, and 77.04° Miller planes of the hexagonal wurtzite crystal phase, which has a polycrystalline nature [29]. The statistics provided by Wang, R. H. et al. [30] and Aydin et al. [31] for pure ZnO nanoparticles closely matched the observed diffraction peaks. The (101) plane was shown by the XRD patterns to be the favored orientation for growth. The hexagonal crystal structure of Nd_2_O_3_’s 100, 002, 101, 102, 110, 103, and 201 Miller planes are responsible for the peaks at 2θ values of 26.90°, 29.85°, 30.89°, 40.52°, 47.55°, 53.58°, and 57.66°, respectively [32]. Additionally, Figure 3b,c displays the XRD pattern for the produced ZnO/Nd_2_O_3_. The graphs show that the material’s XRD pattern is a mixture of XRD peaks from separate ZnO and Nd_2_O_3_ nanomaterials, with lower intensities, suggesting that ZnO and Nd_2_O_3_ were successfully bound to create heterostructures [33]. The occurrence of Nd_2_O_3_ peaks in addition to ZnO peaks shows how Nd_2_O_3_ is associated with the ZnO surface. As seen in Figure 3, the intensities of the diffracted peaks decreased as the Nd concentration increased to 5 mol%, indicating a decrease in the sample’s crystallization. No peaks from other impurities were found, suggesting that Nd_2_O_3_ are present on the ZnO surface but were not integrated into the ZnO lattice [34]. Zn^2+^ and Nd^3+^ have significantly different ionic sizes, which is probably the cause of this drop in crystallinity [35]. Additionally, all of the determined structural characteristics for both pure and modified tribocatalysts are shown in Table 1. The XRD results demonstrate that the addition of Nd_2_O_3_ had no appreciable impact on the crystal size. Nevertheless, when the particles were altered, the ZnO peaks’ intensity decreased. The average crystallite size decreased as the neodymium oxide concentration rose, indicating that the crystalline lattice stayed mostly stable. Table 1 displays the structural properties of the pure, 2 and 5 mol% neodymium oxide-modified sol–gel samples. It was discovered that the unit cell characteristics of the pure ZnO and the Nd_2_O_3_-modified samples were remarkably similar. In the calculations, tensile strain was denoted by positive values. The strain in the sol–gel-derived samples was tensile, and the modified particles showed a larger magnitude of tensile strain than the pure samples, according to the estimates in Table 1.

XPS was employed to investigate the surface composition and oxidation states of elements in the ZnO/Nd_2_O_3_ (0, 1, 2 and 5 mol%) samples before and after tribocatalytic testing. The analysis focused on the Nd 3d and O 1s core levels, as neodymium is expected to modify the ZnO surface, while oxygen states reveal the presence of lattice and non-lattice (defective) species. The Zn 2p core level exhibited no significant variations and remained consistent with the values reported for pure ZnO [36].

**Figure 4:**
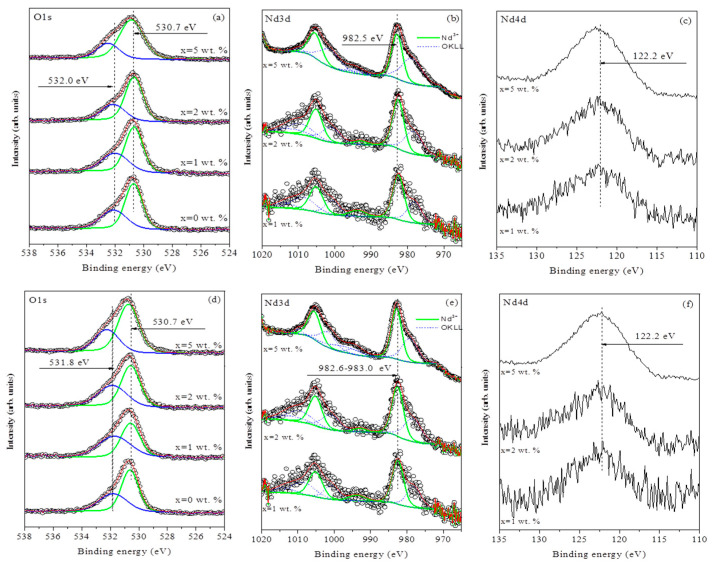
High-resolution X-ray photoelectron spectra of (**a**) O 1s, (**b**) Nd 3d and (**c**) Nd 4d core levels before tribocatalytic testing and high-resolution XP spectra of (**d**) O 1s, (**e**) Nd 3d and (**f**) Nd 4d core levels after tribocatalytic testing for ZnO/Nd_2_O_3_ samples (0, 1, 2 and 5 mol%).

Figure 4 presents high-resolution XPS spectra of (a) O 1s, (b) Nd 3d, and (c) Nd 4d regions for the samples before tribocatalytic testing, arranged in order of increasing neodymium content. The O 1s spectra (Figure 4a) were fitted with two components: the main peak at ~530.7 eV, attributed to lattice oxygen in Zn–O bonds, and a higher-binding-energy component at ~532.0 eV corresponding to non-lattice oxygen species such as oxygen vacancies, hydroxyl groups, or adsorbed water.

The Nd 3d spectra (Figure 4b) exhibited characteristic doublet peaks at ~982.5 eV (Nd 3d_5_/_2_) and ~1005.1 eV (Nd 3d_3_/_2_), consistent with Nd^3+^ oxidation state [37]. Because the O KLL Auger peak (~980 eV) partially overlaps the Nd 3d line shape, an additional verification was performed using the Nd 4d region (Figure 4c). The single symmetric peak centered at 122.2 eV confirmed the exclusive presence of Nd^3+^ ions [38].

Table 2 summarizes the surface atomic concentrations, binding energies, and oxidation states for the ZnO/Nd_2_O_3_ samples before and after tribocatalysis. The surface Nd concentration increases with nominal doping level, while the proportion of non-lattice oxygen remains relatively constant within experimental error, suggesting that low-level Nd_2_O_3_ modification does not significantly disturb the ZnO surface chemistry.

The same set of samples was analyzed after tribocatalytic degradation tests. Figure 4 also shows high-resolution spectra of (d) O 1s, (e) Nd 3d, and (f) Nd 4d regions after tribocatalysis. As before, the O 1s spectra (Figure 4e) were deconvoluted into two components: the lattice oxygen peak at ~530.7 eV, which remained unchanged, and the non-lattice oxygen peak at ~531.8 eV. The minor binding-energy shift toward lower values is negligible and likely arises from curve-fitting uncertainty.

The Nd 3d spectra (Figure 4e) again display Nd^3+^ signals (Nd 3d_5_/_2_ ≈ 982.6 eV), indicating that neodymium preserves its oxidation state during tribocatalysis. The Nd 4d spectra (Figure 4f) retain a single component at 122.2 eV, confirming the chemical stability of neodymium under the reaction conditions.

Table 2 also presents the surface atomic concentrations after tribocatalysis. The Nd^3+^ content follows the same trend as before testing, demonstrating that the dopant remains anchored to the ZnO surface and does not undergo dissolution or reduction. A slight decrease in the proportion of non-lattice oxygen species was observed, which could result from partial filling of oxygen vacancies or surface reconstruction during tribocatalytic operation.

The XPS data reveal that neodymium exists exclusively as Nd^3+^ in both pre- and post-reaction samples, confirming the chemical stability of the modifier and the robustness of the ZnO/Nd_2_O_3_ interface under tribocatalytic conditions. The observed non-lattice oxygen component (~531.8–532 eV) corresponds to oxygen vacancies and low-coordination sites that serve as active centers for reactive oxygen species (ROS) generation. The slight decrease in this component after tribocatalysis indicates partial healing of surface defects as they participate in redox reactions.

Importantly, the constancy of Zn^2+^ and Nd^3+^ states together with the marginal changes in surface composition suggest that the enhanced tribocatalytic activity of Nd-modified ZnO originates not from chemical transformation but from improved charge separation and defect stabilization at the ZnO/Nd_2_O_3_ heterointerface. These findings directly support the proposed tribocatalytic mechanism, in which Nd^3+^ domains act as electron sinks while oxygen vacancies facilitate ROS formation. Thus, the XPS results provide solid evidence that Nd doping enhances the defect-mediated charge-transfer processes responsible for the superior tribocatalytic degradation of doxycycline.

**Figure 5:**
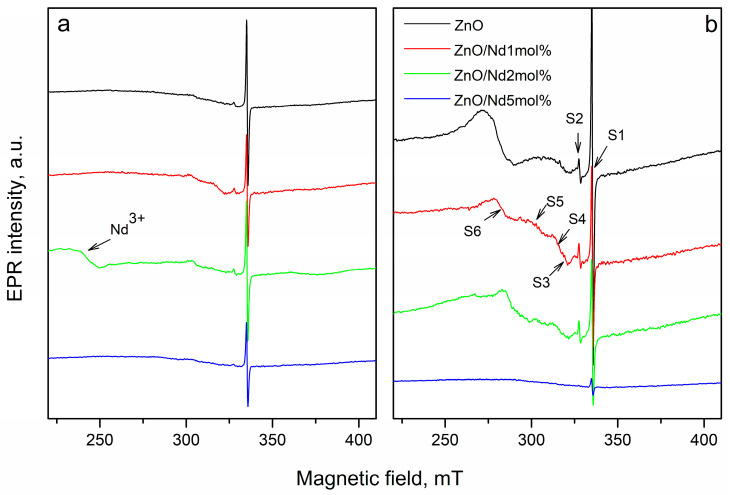
The X-band EPR spectra recorded at room temperature of: (**a**) the pure ZnO NPs and 1, 2 and 5 mol% Nd_2_O_3_-doped ZnO fresh samples, and (**b**) the pure ZnO flowers and 1, 2 and 5 mol% Nd_2_O_3_-doped ZnO samples after tribocatalytic treatment.

EPR study of Nd_2_О_3_-doped ZnO nanostructures was provided to determine the influence of different molar contents of the doping agent on the formation of different defects before and after tribocatalytic treatment. The X-band EPR spectra detected in the undoped ZnO and ZnO doped with Nd_2_О_3_ in different amounts (1, 2 and 5 mol%) before and after tribocatalysis are shown in Figure 5. All samples show an intensive EPR line at g = 1.956 (denoted as S1 in Figure 5), which can be associated with so-called shallow effective mass donor (SD) center in ZnO crystals [39]. This signal probably is associated with Zn-related defects [40,41].

**Figure 6:**
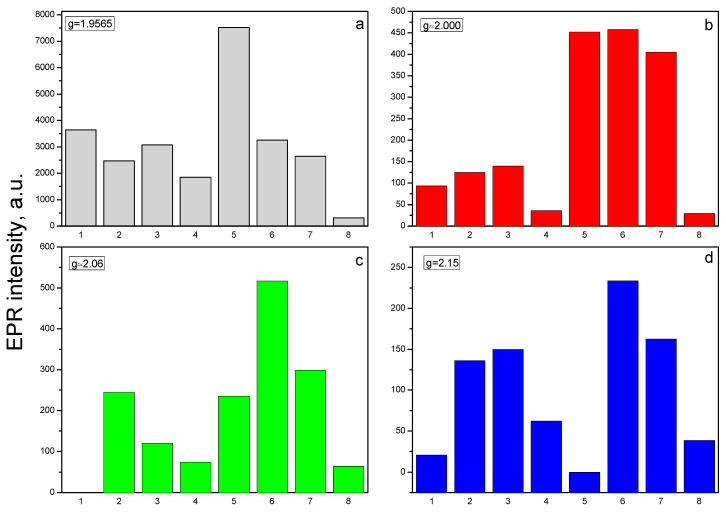
EPR intensity of recorded signals correspond to various g-factors: (**a**) 1.9565, (**b**) 2.00, (**c**) 2.06, and (**d**) 2.15 for individual samples (1–pure ZnO, 2–1 mol% Nd_2_O_3/_ZnO, 3–2 mol% Nd_2_O_3_/ZnO, 4–5 mol% Nd_2_O_3_/ZnO before tribocatalysis; 5–pure ZnO, 6–1 mol% Nd_2_O_3_/ZnO, 7–2 mol% Nd_2_O_3_/ZnO, 8–5 mol% Nd_2_O_3_/ZnO after tribocatalysis).

Some authors related this peak to oxygen vacancy [42]. The study shows that the intensity of S1 depends on the concentration of neodymium (Figure 6a). It was observed, that S1 intensity decreased after doping with Nd_2_O_3_. The possible reason for the reduction in Zn-related defects is the substitution of Zn^2+^ ions with Nd^3+^ ions. In result, cationic vacancies could be created due to the electrical neutrality of ZnO crystal on the follow mechanism: 3Zn^2+^ → 2Nd^3+^ + V _Zn_^2+^.

After mechanochemical activation of the pure ZnO and Nd_2_O_3_-modified ZnO, the number of these defects increases significantly, with the exception of the sample doped with Nd_2_O_3_ in (Figure 6a). The second less intensive EPR signal (S2) is detected at g- value 2.00. According to literature data, the signal with a g-factor close to the free electron 2.0023 in ZnO refers to singly ionized oxygen vacancies (V_0_^+^) [43,44]. In agreement with the above mechanism, more oxygen vacancies (V_0_^+^) are observed in neodymium modified ZnO. In Figure 6b is given the intensity of peak associated with V_0_^+^. The amount of oxygen vacancies in the Nd_2_O_3_ doped ZnO NPs is more than that in the pure ZnO, more significantly in the sample doped with 2 mol%. This result is coincided with work of Hammed et al. [45]. After tribocatalytic treatment amount of oxygen vacancies significantly increase (Figure 6b).

**Figure 7:**
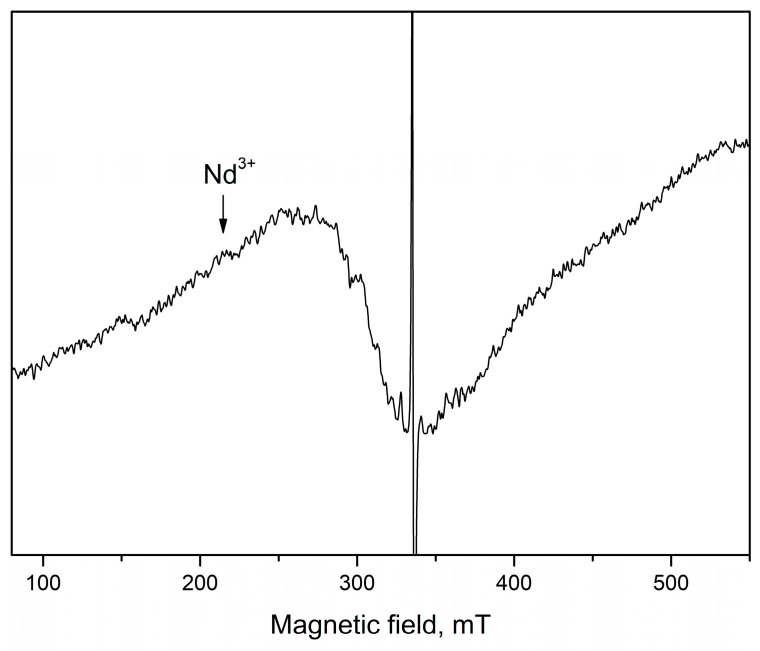
X-band EPR spectrum of the 5 mol% Nd_2_O_3_/ZnO fresh sample recorded at room temperature.

A superposition of several signals with close g-factors were observed in all samples. This complicates the precise identification of some of the detected signals. In the range of magnetic field from 285 mT to 325 mT, three signals at g = 2.05 (S3), at g = 2.07 (S4) and at g = 2.15 (S5) were recorded. The recorded S3 and S4 signals can be assigned to OH^•^ radicals adsorbed on the catalyst surface or could arise from O_2_^−^ species formed from traces of oxygen being present in the system, belonging to ROS species [46,47,48,49]. Since it is difficult to determine the exact intensity of S3 and S4 signals due to their significant overlap, Figure 6c shows the total average g = 2.06. The results show that after tribocatalytic treatment amount of ROS significantly increase in ZnO/Nd_2_O_3_samples (Figure 6c). According to literature, the signal recorded at g = 2.15 probably is associated with Zn vacancies [50]. After modifying of ZnO with neodymium the intensity of S5 signal increased, and after tribocatalysis it additionally increased (Figure 6d). An exception to the obtained results is observed for the sample doped with 5 mol% Nd_2_O_3_. In it, the number of recorded defects in the crystal structure and the number of surface radicals are significantly lower compared to the other samples. In addition, the tribocatalytic treatment of the ZnO/Nd_2_O_3_ 5 mol% sample leads to a decrease in the intensities of the detected signals. The EPR line with g = 2.71 was observed only in ZnO/Nd_2_O_3_ (2 mol%) before tribocatalysis. According to the literature, neodymium paramagnetic centers Nd^3+^ are recorded at g-factor values g_‖_ ≈ 1.3. and g_⊥_≈ 3 [28]. In the spectrum of ZnO/Nd_2_O_3_ (5 mol%) before tribocatalysis, a weakly intense line with g ≈ 3 corresponding to Nd^3+^ is also observed (Figure 6). After tribocatalytic treatment the line attributed to Nd^3+^ not recorded, unlike XPS study. This loss of EPR visibility likely arises from substantial line broadening and/or very fast spin relaxation induced by tribocatalysis (e.g., increased defects, enhanced carrier concentration, adsorbates), which renders the resonance too broad and shallow and therefore invisible in X-band CW EPR (Figure 7). In the ZnO/Nd_2_O_3_ (1 mol%) samples the Nd^3+^ EPR line we are not detected, probably because of small Nd_2_O_3_ quantity. In result of tribocatalytic treatment a new additional line at g = 2.3 with unknown nature was appeared. Having in mind the g value it could be some metal ion.

The EPR spin-trapping technique was employed to investigate free radicals generated during the catalytic degradation of doxycycline in contaminated water using Nd_2_O_3_-doped ZnO. Stable radicals in substances can be directly examined by EPR (as demonstrated earlier in this study). However, when radicals are short-lived, they are detected using so-called spin traps. In this work, 5,5-dimethyl-1-pyrroline-N-oxide (DMPO) was used as the spin-trapping agent. The experiment was carried out directly in the cavity of the EPR spectrometer to ensure that all short-lived species were recorded. The obtained results revealed, two distinct EPR spectral profiles of DMPO-trapped radicals corresponding to ^•^OH and ^•^O_2_^−^ species. Under UVC irradiation, an EPR spectrum consisting of two quartets with intensity ratios of 1:2:2:1 and 1:1:1:1 was appeared. These signals are characteristics of DMPO-OH and DMPO-O_2_- spin adducts, respectively [51].

For all catalytic materials tested in this study, the DMPO-OH signal was more intense (approximately 2.6 times) than the DMPO-O_2_- signal, indicating that ^•^OH radicals play a dominant role in the catalytic degradation of doxycycline. These observations are in good agreement with literature reports, which suggest that ^•^OH radicals and h^+^ are the main active species responsible for the photocatalytic degradation of doxycycline, whereas ^•^O_2_^−^ radicals play only a minor role in the photoreaction [52]. Regarding the influence of the doping agent on the amount of photogenerated radicals, the results showed that increasing of neodymium content, led to a decrease in the intensity of both recorded signals. The reduction in ^•^OH radicals is 1.4 times after modification with 1 mol % Nd_2_O_3_ and 1.6 times at 2 mol % Nd_2_O_3_/ZnO, a similar reduction is observed in ^•^O_2_^−^ radicals accord to pure ZnO. The observed decrease in ROS concentration with increasing Nd content is likely associated with the enhanced participation of these species in the degradation process of the pollutant. This suggests that higher Nd doping facilitates more effective utilization of reactive oxygen species in redox reactions, leading to their faster consumption during the catalytic degradation of doxycycline.

The EPR investigations of Nd_2_O_3_-doped ZnO nanostructures revealed that Nd incorporation significantly affects the defect structure and reactive species formation. Doping with Nd^3+^ ions reduces Zn-related defects while promoting the generation of oxygen vacancies, which act as active centers for catalytic processes. After tribocatalytic treatment, both the number of defects and the amount of reactive oxygen species (ROS) markedly increase, particularly in the ZnO/Nd_2_O_3_ samples, indicating enhanced surface reactivity. EPR spin-trapping experiments confirmed that ^•^OH radicals are the dominant reactive species during doxycycline degradation, while ^•^O_2_^−^ radicals play a secondary role. Overall, Nd doping and tribocatalytic activation synergistically modify the electronic and defect structure of ZnO, improving its catalytic performance through controlled ROS generation.

**Figure 8:**
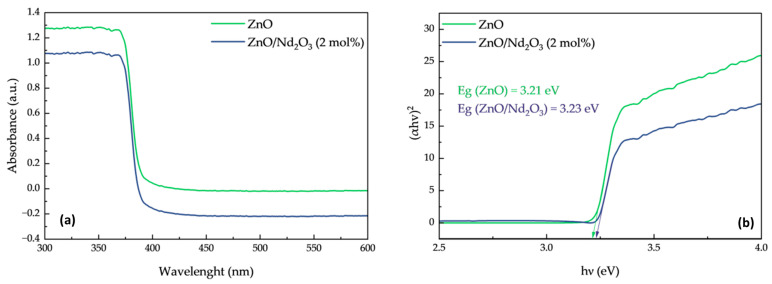
(**a**) UV–visible absorption spectra of ZnO and ZnO/Nd_2_O_3_ (2 mol%) nanoflowers and (**b**) Tauc plots of (αhν)^2^ versus photon energy for band gap estimation.

The optical band gap energies (E_g_) were estimated using Tauc plots of (αhν)^2^ versus hν (Figure 8). The calculated band gap values were 3.21 eV for ZnO and 3.23 eV for ZnO/Nd_2_O_3_ (2 mol%), showing only a negligible difference. This confirms that the observed enhancement in tribocatalytic degradation activity upon Nd_2_O_3_ incorporation is not due to band gap narrowing or improved visible-light absorption.

Instead, the improved performance is attributed to the formation of ZnO/Nd_2_O_3_ heterointerfaces, which facilitate charge separation and suppress electron–hole recombination under triboelectric excitation. Nd_2_O_3_ acts as an electron sink and provides additional active sites for oxygen adsorption, thereby promoting the generation of reactive oxygen species (ROS). The UV–Vis results thus support the conclusion that the catalytic improvement arises from interfacial and electronic effects rather than intrinsic band structure modification.

### 2.2. Tribocatalytic Degradation of Doxycycline at Three Different Stirring Speeds

**Figure 9:**
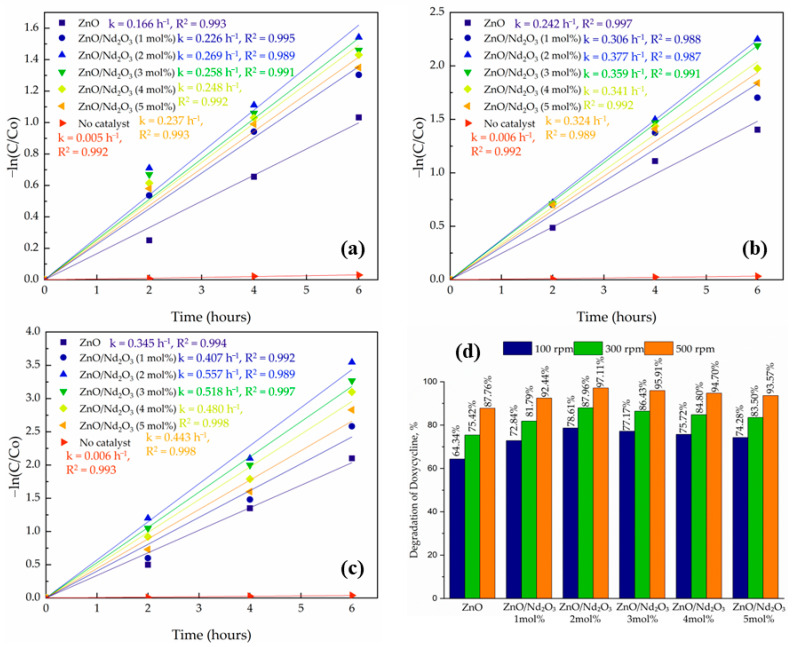
Kinetic plots of –ln(C/C_0_) versus time for ZnO and ZnO/Nd_2_O_3_ nanoflowers at (**a**) 100 rpm, (**b**) 300 rpm, and (**c**) 500 rpm, and (**d**) comparison of degradation efficiencies after 6 h.

The tribocatalytic degradation of doxycycline was studied without catalysts and with ZnO and ZnO/Nd_2_O_3_ nanoflowers containing 1 and 2 mol% Nd_2_O_3_. The reaction progress was monitored by plotting –ln(C/C_0_) versus time, which showed good linearity in all cases, indicating that the degradation follows pseudo-first-order kinetics (Figure 9). The importance of the catalyst in the friction process is highlighted by the fact that a control experiment without a tribocatalyst showed very little degradation (about 3%).

The influence of stirring speed was also systematically examined at 100, 300 and 500 rpm for all catalysts. In every case, the degradation efficiency increased with increasing rpm, following the order 500 > 300 > 100 rpm (Figure 9). At 100 rpm, only a slower degradation was observed (e.g., ZnO/Nd_2_O_3_ (2 mol%) achieved 78.61%, whereas at 500 rpm, the same sample reached 97.11%, demonstrating the strong dependence of tribocatalytic performance on stirring intensity. This enhancement can be rationalized by the role of stirring speed in both charge generation and mass transfer: higher rpm increases the frequency and intensity of collisions between the stirring rod, catalyst particles, and vessel walls, generating a larger flux of triboelectric charges. At the same time, stronger mixing reduces particle agglomeration and accelerates the transport of oxygen and DOX molecules to the catalyst surface. Together, these processes intensify reactive oxygen species (ROS) production, thereby accelerating degradation.

The superior performance of the nanoflowers can be attributed to the interfacial interaction between ZnO and Nd_2_O_3_. Formation of ZnO/Nd_2_O_3_ heterojunctions facilitates the separation of tribo-induced charge carriers, as electrons generated on ZnO can migrate to Nd_2_O_3_, thereby reducing recombination. In addition, Nd_2_O_3_ provides extra active sites for oxygen adsorption and subsequent ROS generation. The synergistic effect of these processes explains the marked improvement in catalytic efficiency compared to pristine ZnO. It should be noted that additional experiments conducted with higher Nd_2_O_3_ contents (3, 4, and 5 mol%) resulted in significantly lower tribocatalytic activity; this decrease is attributed to excessive Nd_2_O_3_ loading, which partially covers active ZnO surfaces, introduces deep recombination traps, and disrupts the optimal ZnO/Nd_2_O_3_ interfacial ratio required for efficient charge separation, as similarly reported for rare-earth-modified ZnO systems [53,54,55].

Among all systems, the ZnO/Nd_2_O_3_ (2 mol%) composite at 500 rpm exhibited the highest degradation rate and kinetic constant, confirming that an optimized proportion of Nd_2_O_3_ enhances interfacial charge transfer and ROS generation without introducing excessive carrier trapping. These findings are consistent with previous reports on oxide–rare-earth catalysts, where efficient charge separation and surface reactivity are the key drivers of improved catalytic activity [56].

Overall, the results confirm that both catalyst design (Nd_2_O_3_ content) and operational parameters (stirring speed) strongly affect tribocatalytic efficiency. ZnO/Nd_2_O_3_ (2 mol%) nanoflowers under optimized stirring conditions demonstrate the most promising performance, highlighting their potential application in wastewater remediation under dark, mechanically driven catalytic environments.

### 2.3. Tribocatalytic Degradation of Doxycycline at Two Types of Magnetic Rods

**Figure 10:**
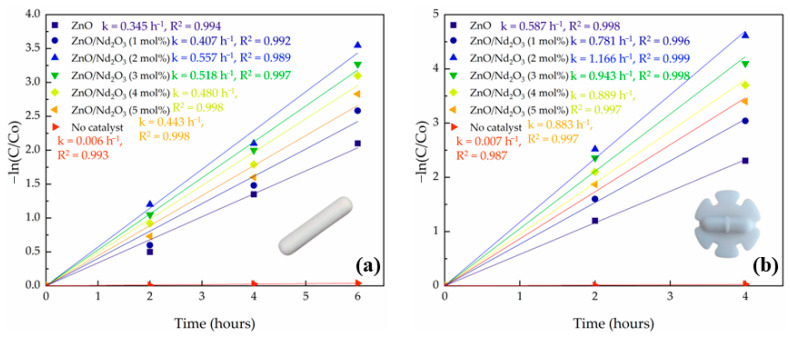
Kinetic plots of –ln(C/C_0_) versus time for ZnO and ZnO/Nd_2_O_3_ nanoflowers at 500 rpm using (**a**) a cylindrical stirring rod and (**b**) a flower-shaped stirring rod.

The geometry of the magnetic stirring rod was found to exert a pronounced influence on the tribocatalytic degradation of doxycycline. Figure 10 shows the kinetic plots of –ln(C/C_0_) versus reaction time for ZnO modified with Nd_2_O_3_ (0, 1, 2, 3, 4 and 5 mol%) using a conventional cylindrical stirring bar and a flower-shaped bar. In all cases, the plots retained linearity, confirming pseudo-first-order kinetics; however, the slopes were consistently steeper when the flower-shaped bar was employed, indicating larger apparent rate constants and significantly faster degradation. For instance, ZnO degradation increased from 87.76% with the normal bar to 90.04% with the flower bar, while ZnO/Nd_2_O_3_ (2 mol%) reached as high as 99.00%. Notably, when the flower-shaped rod was used, complete degradation was achieved within 4 h, compared to 6 h with the conventional rod. Further tests using higher Nd_2_O_3_ concentrations (3, 4, and 5 mol%) with flower-like rods similarly showed noticeably reduced tribocatalytic activity. Excessive Nd_2_O_3_ loading was blamed for this decline because it created deep recombination traps and interfered with the ideal ZnO/Nd_2_O_3_ interfacial ratio [53,54]. The data is summarized in Table 3. On the other hand, very little degradation (roughly 3%) was seen in a control experiment without a tribocatalyst, underscoring the catalyst’s significance in the friction process.

To rationalize these observations, the hydrodynamic behavior induced by each stirrer geometry was quantitatively assessed using the impeller Reynolds number [55,56]. For water at 25 °C (ρ = 997 kg m^−3^, μ = 8.9 × 10^−4^ Pa.s) and a stirring speed of 500 rpm (8.33 s^−1^), the calculated Reynolds number for the flower-shaped stirrer (d = 2.5 cm) was ap-proximately 5.8 × 10^3^, corresponding to the transitional-to-turbulent flow regime. In contrast, the cylindrical bar (d = 0.6 cm) yielded a much lower Reynolds number of about 3.3 × 10^2^. These values confirm that the flower-shaped geometry produces significantly stronger mixing, secondary circulation, and localized turbulence.

The enhanced activity of the flower-shaped bar can be explained by two key factors: hydrodynamics and triboelectric charge generation. First, the complex geometry of the flower rod produces stronger turbulence and secondary flows compared to the smoother cylindrical bar. This improves catalyst dispersion, prevents particle sedimentation, and reduces the thickness of the liquid boundary layer, thereby accelerating mass transfer of both DOX molecules and dissolved oxygen to the catalyst surface. Second, the lobed and edged design of the flower bar increases the effective contact area and generates more frequent and forceful collisions between the stirring rod, catalyst particles, and reactor walls. These intensified frictional interactions enhance the triboelectric effect, producing a higher flux of charge carriers that subsequently react with dissolved oxygen and water to generate reactive oxygen species (ROS).

Importantly, while both bar geometries showed the same activity order of ZnO < ZnO/Nd_2_O_3_ (1 mol%) < ZnO/Nd_2_O_3_ (2 mol%), the absolute degradation efficiencies and rate constants were significantly higher with the flower-shaped rod. This indicates that the improved hydrodynamic and triboelectric conditions provided by the flower geometry amplify the intrinsic advantages of the ZnO/Nd_2_O_3_ nanoflowers, leading to the most efficient degradation in the ZnO/Nd_2_O_3_ (2 mol%) and flower bar system.

These results highlight that, in addition to catalyst design, reactor configuration—specifically the geometry of the stirring rod—plays a critical role in tribocatalytic performance. Optimizing both factors in tandem offers a powerful route to enhance the removal efficiency of pharmaceutical pollutants under dark, mechanically driven catalytic conditions.

### 2.4. Tribocatalytic Degradation of Doxycycline with Two Different Types of Beakers

In addition to catalyst composition, stirring geometry, and stirring speed, the material of the reaction vessel was found to significantly influence tribocatalytic efficiency. Comparative experiments were carried out in glass and PTFE beakers at 500 rpm using both normal and flower-shaped magnetic stirring rods. The results are summarized in Table 3 (rate constants, R^2^ values, and final degradation efficiencies).

Degradation efficiencies were consistently higher in PTFE vessels, with the flower rod providing the strongest enhancement. Under these optimized conditions, ZnO and ZnO/Nd_2_O_3_ (1 mol%) achieved complete degradation of doxycycline within 4 h, while the ZnO/Nd_2_O_3_ (2 mol%) composite required only 2 h. The kinetic constants (Table 4) confirm this trend, with the ZnO/Nd_2_O_3_ (2 mol%) + PTFE/flower rod system exhibiting the highest k value and excellent linearity, consistent with rapid pseudo-first-order degradation.

The superior performance of the PTFE beaker can be attributed to its triboelectric properties. As one of the most electronegative materials in the triboelectric series, PTFE readily acquires negative charges upon frictional contact with catalyst particles or the stirring rod [57]. This enhances charge separation during tribocatalysis, increasing the availability of free electrons and holes that drive the generation of reactive oxygen species (ROS). In contrast, glass surfaces are less efficient in producing such charges, leading to slower kinetics and lower degradation percentages.

The effect is further amplified by the flower-shaped rod, whose geometry promotes stronger turbulence and more frequent collisions between catalyst particles and the vessel wall. This combination maximizes both triboelectric charge generation and oxygen activation, resulting in the fastest degradation observed in this study. The finding that ZnO/Nd_2_O_3_ (2 mol%) achieves complete degradation within only 2 h highlights the synergistic role of reactor wall material, stirring geometry, and catalyst composition in optimizing tribocatalytic processes.

### 2.5. Plausible Mechanism of Tribocatalysis

**Figure 11:**
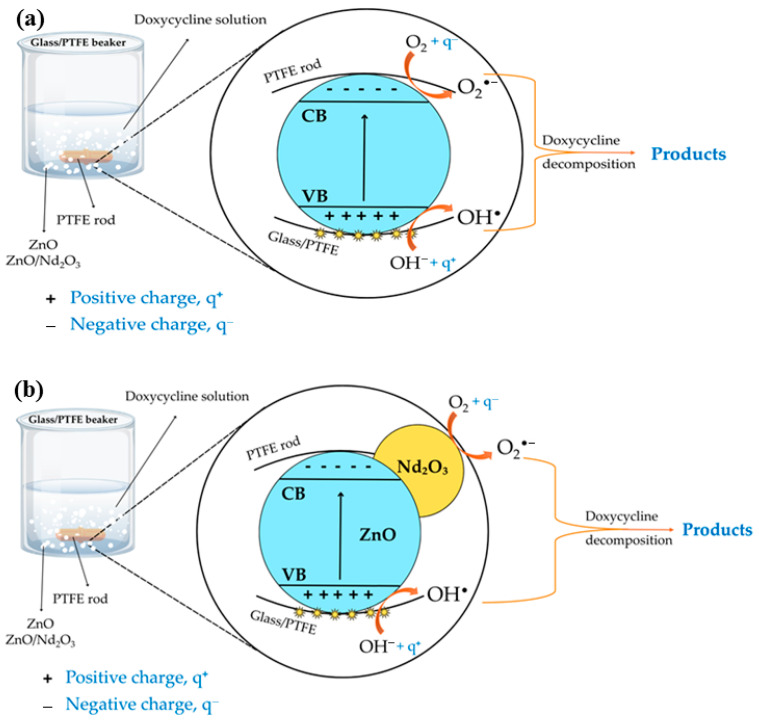
Plausible mechanism of tribocatalytic degradation of doxycycline (DOX) by (**a**) ZnO and (**b**) ZnO/Nd_2_O_3_ flowers.

Based on the experimental findings, a plausible mechanism for the tribocatalytic degradation of doxycycline by ZnO and ZnO/Nd_2_O_3_ nanoflowers is proposed (Figure 11). When ZnO absorbs mechanical energy during friction, electrons are represented by excited e^–^, and holes are represented by the resulting h^+^. When oxygen molecules interact with electrons during the drug’s degradation, O^2−^ superoxide radicals are produced. The holes transform into hydroxyl radicals, or OH^•^, after interacting with OH^–^. The effective electron-hole separation across the ZnO/Nd_2_O_3_ interface and the increased production of O^2−^ and OH^•^ radicals may be responsible for the increased activity of the neodymium-modified ZnO sample. The increased efficiency is a result of the higher adsorption of hydroxyl ions onto the ZnO surface and the increased number of oxygen vacancies in the Nd-modified ZnO due to the different charge and electronegativity of zinc and neodymium ions [51,58]. The formation of OH^•–^ is facilitated by the reaction between the holes and OH. Strong, non-selective oxidants like hydroxyl radicals and other tribogenerated active species break down organic contaminants on the surface of neodymium-modified ZnO [59,60]. ZnO modified with the neodymium oxide phase exhibits higher catalytic efficiency, probably because tribogenerated charge recombination is suppressed. The neodymium phase is beneficial because it traps electrons, prevents electron-hole recombination reactions, and produces more superoxide and hydroxyl radicals, all of which hasten the breakdown of pollutants.

In addition to catalyst composition, reactor configuration strongly influences tribocatalytic efficiency. The flower-shaped stirring rod enhances turbulence and increases collision frequency, thereby intensifying charge generation. Similarly, the use of PTFE beakers further promotes electron accumulation due to PTFE’s high electronegativity in the triboelectric series, providing a more favorable environment for charge transfer. Under these synergistic conditions, ROS production is maximized, explaining the rapid and complete degradation of doxycycline observed with ZnO/Nd_2_O_3_ (2 mol%) in a PTFE beaker using a flower-shaped rod [61].

**Figure 12:**
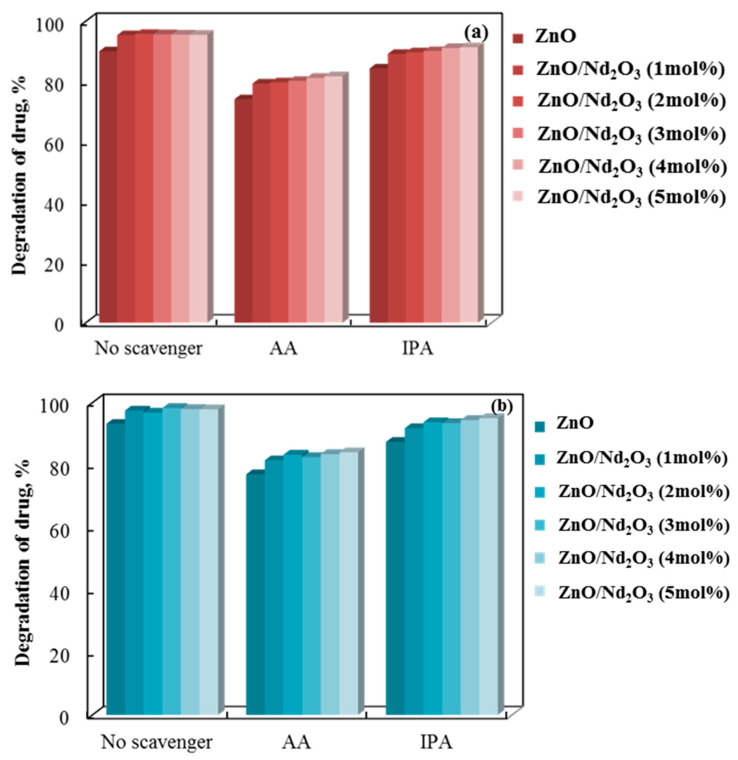
Effects of scavengers on the tribocatalytic breakdown of doxycycline in a PTFE beaker using (**a**) cylindrical (6 h process) and (**b**) flower-like (4 h process) rods.

The involvement of superoxide and hydroxyl radicals is demonstrated by a radical scavenger assay we performed. Figure 12 shows the information. Superoxide and hydroxyl radicals’ roles in the breakdown of doxycycline were quantified through the addition of ascorbic acid (AA) and isopropyl alcohol (IPA) scavengers, which capture the corresponding reactive species [62,63].

Figure 12 illustrates the comparable effects of adding AA and IPA to the three tribocatalyst systems, with the former exhibiting a more pronounced inhibition. This implies that the doxycycline tribo-degradation rate is more affected by the superoxide radical.

**Figure 13:**
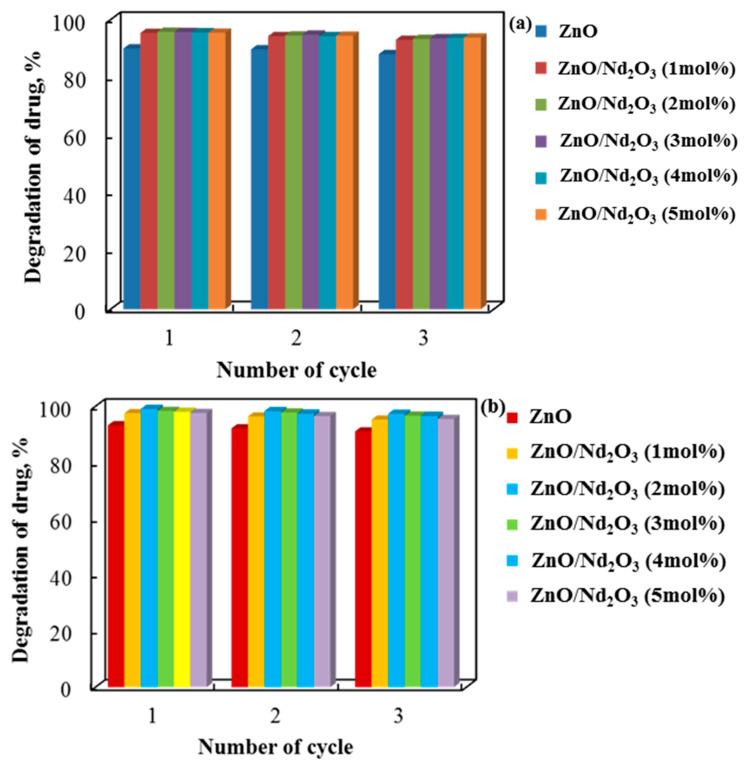
Doxycycline decolorization rate in a PTFE beaker using (**a**) cylindrical (6 h tribocatalysis) and (**b**) flower-like (4 h tribocatalysis) rods for three consecutive cycles.

Figure 13 shows a three-cycle study on the recyclability of tribocatalysts composed of pure and neodymium-modified zinc oxide with cylindrical and flower-like rods. The tribocatalytic breakdown of doxycycline dropped by about 2% for all catalyst types after three cycles in distilled water, suggesting that the tribocatalysts’ catalytic performance declined slightly with each cycle. The drug decomposition cycle of the sol–gel samples was found to be stable in spite of this decline. These findings show that they can be used repeatedly to break down paracetamol. Although ZnO/2 mol% shows a slight decrease with repeated use, Nd_2_O_3_ is the most stable and effective catalyst over many cycles.

## 3. Materials and Methods

### 3.1. Materials

Zinc nitrate hexahydrate (Zn(NO_3_)_2_·6H_2_O, ≥98%), sodium hydroxide (NaOH, ≥98%), and neodymium (III) oxide (Nd_2_O_3_, ≥99.9%) were purchased from Sigma-Aldrich and used without further purification. Deionized water was used for all preparations.

### 3.2. Synthesis Procedure of Pure ZnO Nanoflowers

Zinc oxide nanoflowers were synthesized via a sol–gel method. Zinc nitrate hexahydrate (3.0 g, 10.1 mmol) was dissolved in 80 mL of deionized water under constant magnetic stirring. Separately, sodium hydroxide (1.6 g, 40 mmol) was dissolved in 80 mL of deionized water. The NaOH solution was added dropwise into the zinc nitrate solution with continuous stirring, and the resulting mixture was transferred into a round-bottom flask and maintained at 80 °C for 6 h. The resulting white precipitate was collected by vacuum filtration, thoroughly washed several times with deionized water to remove residual ions, and dried at 100 °C for 6 h. The dried precursor was then calcined at 350 °C for 3 h in air to obtain ZnO nanoflowers.

### 3.3. Synthesis Procedure of ZnO/ Nd_2_O_3_ Nanoflowers

ZnO/ Nd_2_O_3_ nanoflowers were prepared following the same procedure as described above, with the incorporation of neodymium oxide (Nd_2_O_3_). Prior to the addition of NaOH, a predetermined amount of Nd_2_O_3_ corresponding to 1 mol% and 2 mol% relative to Zn^2+^ was dispersed in the zinc nitrate solution by ultrasonication for 30 min to ensure homogeneous distribution. After sonication, the NaOH solution was added dropwise under stirring, and the mixture was subjected to the same precipitation, filtration, washing, drying, and calcination steps as used for pure ZnO. The obtained products were designated as ZnO/Nd_2_O_3_ (1 mol%) and ZnO/Nd_2_O_3_ (2 mol%), respectively.

### 3.4. Methods

The morphology and microstructure of the synthesized samples were examined using a scanning electron microscope (SEM, JSM-5510, JEOL, Krefeld, Germany). Elemental analysis and chemical characterization were carried out by energy-dispersive X-ray spectroscopy (EDS, Quantax 200 detector, Bruker Resolution 126 eV, Berlin, Germany), and elemental mapping was used to confirm the distribution of Zn, O, and Nd. Scherrer’s equation was used to estimate the average crystallite sizes. Rietveld analysis was done with PowderCell [64], and the March-Dollase texturing model [65] was utilized to investigate whether the pure and Nd-modified ZnO samples showed signs of preferential orientation. X-ray photoelectron spectroscopy (XPS) measurements were performed using an ESCALAB MkII (VG Scientific, now Thermo Fisher Scientific, Waltham, MA, USA) electron spectrometer at a base pressure of 5 × 10^−10^ mbar in the analysis chamber (rising to 2 × 10^−9^ mbar during measurements). An Al Kα X-ray source (hν = 1486.6 eV) was used for excitation. The pass energy of the hemispherical analyzer was set to 20 eV for O 1s spectra, while for Zn 2p and Nd 3d (recorded simultaneously), a pass energy of 50 eV was employed due to the weak signal intensity resulting from the low neodymium concentration on the surface. The instrumental resolution was about 1.0 eV, as determined from the full width at half maximum (FWHM) of the Ag 3d_5_/_2_ peak. Data analysis was carried out using SpecsLab2 and CasaXPS software (2.3.25PR1., Casa Software Ltd., Tokyo, Japan). Processing of the measured spectra included subtraction of X-ray satellites and Shirley-type background [64]. Peak positions and areas were evaluated by symmetric Gaussian-Lorentzian curve fitting. The relative concentrations of different chemical species were determined by normalizing peak areas to their photoionization cross-sections calculated by Scofield [65]. The problem of peak overlap is critical in the analysis of XP spectra, and the difficulties increase when one of the elements is present in negligible concentrations on the investigated surface. This is the case here, as neodymium is present at only 1–2 at.% on the surface. To address this issue, spectra were corrected by subtracting the previously measured substrate background in the same energy range. In this way, the resulting spectra could be analyzed to determine both the oxidation state and the surface atomic concentration of the minor element, i.e., neodymium.

The catalysts ZnO, ZnO/Nd_2_O_3_ (1, 2 mol%) before and after tribocatalysis are investigated directly in powdered form.

To study short-lived radicals, the so-called spin-trapping technique is applied using spin traps. The spin trap molecule interacts with the short-lived radical, forming so-called spin adducts, which have a longer lifetime and can be detected by EPR spectroscopy. In the present study, DMPO (5,5-dimethyl-1-pyrroline-N-oxide) is used as a spin trap.

For sample preparation, 10 mg of the respective catalyst is added to 10 mL of doxycycline-contaminated water. Then, 0.5 mL of a 100 mM DMPO solution in bidistilled water is added to 0.8 mL of the resulting solution. The sample is transferred into a quartz capillary tube with an outer diameter of 2 mm and irradiated with UV light of wavelength 390 nm directly in the resonator of the EPR spectrometer through a special window. The sample fills the entire volume of the resonator. EPR spectra are registered both during irradiation and immediately after irradiation.

The spectra are recorded in air at room temperature under the following instrumental parameters: modulation frequency 100 kHz, modulation amplitude 0.2 mT, field center 317.968 mT, magnetic field sweep 200 mT, microwave power 5 mW, and time constant 0.1 s.

The EPR spectra in the X-band (9.4 GHz) were recorded as a first derivative of the absorption signal of an JEOL JES-FA 100 EPR. The JEOL spectrometer with a 100-kHz magnetic field modulation was equipped with a standard TE_011_ cylindrical resonator. The set of measurements for identifying paramagnetic species was performed at room temperature. All measurements were equated to the same gain and mass of the studied sample.

UV-Vis spectra were recorded using an Evolution 300 spectrophotometer (Thermo Scientific, Madison, WI, USA), to determine the optical absorption characteristics and to estimate the band gap energies via Tauc plots.

Tribocatalytic activity was evaluated by monitoring the degradation of doxycycline (DOX) under dark conditions. The residual DOX concentration was quantified by UV–Vis spectrophotometry (Thermo Scientific, Madison, WI, USA) at the characteristic absorption maximum of 275 nm.

### 3.5. Tribocatalytic Degradation Experiments

Tribocatalytic degradation tests were carried out using 50 mL aqueous solutions of doxycycline (DOX) prepared in 100 mL beakers. Unless otherwise stated, the initial concentration of DOX was 15 ppm. A fixed catalyst dosage of 50 mg (ZnO or ZnO/Nd_2_O_3_ nanoflowers containing 1 or 2 mol% Nd_2_O_3_) was dispersed into the solution and magnetically stirred at room temperature (23 ± 2 °C) under dark conditions. Prior to initiating the tribocatalytic reaction, the suspensions were agitated for 30 min to establish adsorption–desorption equilibrium between DOX molecules and the catalyst surface.

The reactions were then conducted under different operational parameters, including stirring speeds (100, 300, and 500 rpm), magnetic stirrer geometry (conventional cylindrical bar and flower-shaped bar), and reactor vessel material (glass or PTFE beakers). At specified time intervals, 2 mL aliquots of the reaction suspension were collected in daylight and passed through 0.22 μm membrane filters (Millipore, Sigma Aldrich, Burlington, MA, USA) to remove the catalyst particles before analysis. The residual DOX concentration in the supernatant was determined by UV-Vis spectrophotometry (Evolution 300, Thermo Scientific, Madison, WI, USA) at 275 nm.

The tribocatalytic process is attributed to friction-induced electron transfer occurring at the interfaces between the stirring rod, the catalyst particles, and the reactor wall. The use of PTFE beakers and PTFE-coated stir bars is particularly relevant, as PTFE is highly electronegative in the triboelectric series and can efficiently accumulate negative charges during mechanical contact. This promotes charge separation and enhances the generation of reactive oxygen species (ROS), thereby improving degradation efficiency.

The reactive species that were degrading the doxycycline were investigated using a scavenger test. Ascorbic acid (AA) and isopropyl alcohol (IPA) were used as scavengers to absorb superoxide and hydroxyl radicals, respectively. To identify the specific reactive species responsible for the tribocatalytic degradation of the organic drug (50 mL), six milligrams of each scavenger were used separately.

## 4. Conclusions

In this study, ZnO/Nd_2_O_3_ nanoflowers demonstrated significantly higher tribocatalytic activity than pristine ZnO for doxycycline removal under dark conditions. The reactions followed pseudo-first-order kinetics, with the ZnO/Nd_2_O_3_ (2 mol%) composite exhibiting the highest rate constant and complete degradation within only 2 h under optimized conditions (PTFE beaker, flower-shaped rod, 500 rpm). Performance was strongly influenced by operational parameters: higher stirring speed enhanced charge generation and mass transfer, while the PTFE reactor and flower-shaped stirrer amplified the triboelectric effect, leading to faster degradation.

The physicochemical characterization clarified the origin of this enhanced activity. EPR and XPS analyses confirmed that Nd^3+^ incorporation modifies the defect structure of ZnO, suppressing Zn-related defects while generating additional oxygen vacancies that act as active sites for ROS formation. After tribocatalytic treatment, both defect density and the amount of ROS (^•^OH, ^•^O_2_^−^) increased markedly, confirming their participation in the redox process. Spin-trapping tests verified that ^•^OH radicals are the dominant reactive species, whereas ^•^O_2_^−^ radicals play a secondary role.

The proposed mechanism integrates these observations: friction between the catalyst, stirrer, and reactor wall generates triboelectric charges that excite electrons and holes. Nd_2_O_3_ domains act as electron traps, suppressing recombination and enabling sustained ROS formation, while oxygen vacancies and surface hydroxyl groups serve as active sites for radical generation. The cooperative effects of structural defects, interfacial charge transfer, and reactor-induced triboelectric enhancement collectively explain the outstanding catalytic performance.

This work highlights a general design concept for tribocatalytic systems—integrating defect-engineered oxides with triboelectrically active reactor components—to efficiently convert mechanical energy into chemical reactivity for sustainable antibiotic degradation and water purification.

## Figures and Tables

**Table 1:** The ZnO and ZnO/Nd_2_O_3_ flowers’ XRD patterns were used to calculate their structural characteristics.

Tribocatalysts	Crystallite Size, nm	Parameters, Å Microstrain
ZnO	57.9	a, b = 3.2476; c = 5.2017 1.04 × 10^−3^
ZnO/Nd_2_O_3_, 2 mol%	ZnO: 49.85 Nd_2_O_3_: 39.83	а = b: 3.2473; c = 5.2023 3.53 × 10^−4^
		а = b: 3.8255; c = 6.0170 1.88 × 10^−3^
ZnO/Nd_2_O_3_, 5 mol%	ZnO: 45.33 Nd_2_O_3_: 35.11	а = b: 3.2468; c = 5.2024 6.31 × 10^−4^ а = b: 3.8249; c = 6.0164 1.24 × 10^−3^

**Table 2:** Surface atomic concentrations (at.%), binding energies (eV), and corresponding oxidation states/bond types of elements in ZnO/Nd_2_O_3_ samples (0, 1, 2 and 5 mol%) prior to and after tribocatalytic testing.

	Zn-O (Zn 2p)	Zn-O (O 1s)	Non-Lattice (O 1s)	Nd^3+^ (Nd 3d)
Sample	ZnO before tribocatalytic test			
at.%	53.57	28.79	17.64	-
BE, eV	1021.7	530.7	532.1	-
Sample	ZnO/Nd_2_O_3_ (1mol%) before tribocatalytic test			
at.%	52.22	29.70	17.65	0.44
BE, eV	1021.7	530.7	532.0	982.5
Sample	ZnO/Nd_2_O_3_ (2 mol%) before tribocatalytic test			
at.%	51.61	32.54	14.84	1.01
BE, eV	1021.7	530.7	532.0	982.5
Sample	ZnO/Nd_2_O_3_ (5 mol%) before tribocatalytic test			
at.%	43.48	39.71	13.95	2.86
BE, eV	1021.7	530.8	532.5	982.9
Sample	ZnO after tribocatalytic test			
at.%	54.94	26.58	18.48	-
BE, eV	1021.7	530.7	531.8	-
Sample	ZnO/Nd_2_O_3_ (1 mol%) after tribocatalytic test			
at.%	51.41	23.86	24.33	0.40
BE, eV	1021.7	530.6	531.7	982.6
Sample	ZnO/Nd_2_O_3_ (2 mol%) after tribocatalytic test			
at.%	50.74	27.97	20.49	0.81
BE, eV	1021.7	530.6	531.8	982.6
Sample	ZnO/Nd_2_O_3_ (5 mol%) after tribocatalytic test			
at.%	43.40	35.41	18.24	2.95
BE, eV	1021.7	530.7	532.2	982.9

**Table 3:** Rate constants (k), and degradation percentages of Doxycycline for ZnO and ZnO/Nd_2_O_3_ nanoflowers using cylindrical and flower-shaped stirring rods at 500 rpm.

Sample	Cylindrical Rod		Flower-Shaped Rod	
	k, h^−1^	D, %	k, h^−1^	D, %
ZnO	0.345	87.76	0.587	90.04
ZnO/Nd_2_O_3_ (1 mol%)	0.407	92.44	0.781	95.22
ZnO/Nd_2_O_3_ (2 mol%)	0.557	97.11	0.999	99.00
ZnO/Nd_2_O_3_ (3 mol%)	0.518	95.91	0.943	98.10
ZnO/Nd_2_O_3_ (4 mol%)	0.480	94.70	0.889	97.11
ZnO/Nd_2_O_3_ (5 mol%)	0.443	93.57	0.833	96.15

**Table 4:** Rate constants (k), correlation coefficients (R^2^), degradation percentages, and degradation times of ZnO and ZnO/Nd_2_O_3_ nanoflowers under different reactor conditions (beaker material and stirring rod geometry) at 500 rpm.

Sample	Beaker Material	Rod Geometry	k, h^−1^	R^2^	D %; Time, h
ZnO	Glass	Cylindrical	0.345	0.994	87.76; 6
		Flower-like	0.587	0.998	90.04; 4
	PTFE	Cylindrical	0.405	0.994	90.05; 6
		Flower-like	0.714	0.981	93.23; 4
ZnO/Nd_2_O_3_ (1 mol%)	Glass	Cylindrical	0.407	0.992	92.44; 6
		Flower-like	0.781	0.996	95.22; 4
	PTFE	Cylindrical	0.569	0.979	95.42; 6
		Flower-like	0.982	0.978	97.51; 4
ZnO/Nd_2_O_3_ (2 mol%)	Glass	Cylindrical	0.557	0.989	97.11; 6
		Flower-like	1.166	0.999	99.00; 4
	PTFE	Cylindrical	0.800	0.996	95.82; 6
		Flower-like	1.588	0.998	99.04; 2
ZnO/Nd_2_O_3_(3 mol%)	Glass	Cylindrical	0.518	0.997	95.91, 6
		Flower-like	0.943	0.998	98.10, 4
	PTFE	Cylindrical	0.751	0.992	95.71, 6
		Flower-like	1.280	0.979	98.31, 4
ZnO/Nd_2_O_3_(4 mol%)	Glass	Cylindrical	0.480	0.998	94.70, 6
		Flower-like	0.889	0.997	97.11, 4
	PTFE	Cylindrical	0.690	0.989	95.60, 6
		Flower-like	1.357	0.995	98,00, 4
ZnO/Nd_2_O_3_(5 mol%)	Glass	Cylindrical	0.443	0.998	93.57, 6
		Flower-like	0.833	0.997	96.15, 4
	PTFE	Cylindrical	0.923	0.983	95.50, 6
		Flower-like	1.133	0.994	97.90, 4

## Data Availability

The original contributions presented in this study are included in the article. Further inquiries can be directed to the corresponding author.
